# COMPARING VARIED EFFECTS OF WORK AND PARTICIPATION ON DEVELOPMENT OF OLDER JAPANESE ACROSS GENDERS

**DOI:** 10.1093/geroni/igad104.2390

**Published:** 2023-12-21

**Authors:** Keiko Katagiri

**Affiliations:** Kobe University, Kobe, Hyogo, Japan

## Abstract

Recently, the physical and cognitive status of older Japanese adults has improved on previous generations. Half of 60-year-old Japanese remain in the labor force. With extended life expectancy, improved health, and ability to remain productive into older age, some gerontologists now regard generativity as a developmental task for older adults before becoming frail, rather than for middle-aged people, as previously thought. These changes assume that older adults continue to develop, and this study examines the effects of work and active participation on development of older adults as a potential human resource for society. A 2019 random sampling survey was conducted with Tokyo and Hyogo Prefecture residents; the response rate was 43.0% (N = 1,063, aged 50-74). ANCOVA tests were conducted to gauge sense of community, interest in politics and generativity (all were positively affected by social and civic participation). Older people exhibited a higher sense of community than younger people. Females showed higher generativity and sense of community than males, while males scored higher than females for interest in politics. We observed interaction effects of work and civic participation. Working males in engaging civic activity exhibited greater positive change compared to non-working males with civic activity. Non-working females with active participation demonstrated greater positive change in generativity and interest in politics than working females with activity. This suggests older males enriched their experience by participating in civic activity while older females developed it through work. Both cases suggested that older adults can develop when they experience an unfamiliar diverse world.

